# Effects of Metformin on Spontaneous Ca^2+^ Signals in Cultured Microglia Cells under Normoxic and Hypoxic Conditions

**DOI:** 10.3390/ijms22179493

**Published:** 2021-08-31

**Authors:** Silvija Jankeviciute, Natasa Svirskiene, Gytis Svirskis, Vilmante Borutaite

**Affiliations:** Neuroscience Institute, Lithuanian University of Health Sciences, LT-50161 Kaunas, Lithuania; natasa.svirskiene@lsmuni.lt (N.S.); gytis.svirskis@lsmuni.lt (G.S.); vilmante.borutaite@lsmuni.lt (V.B.)

**Keywords:** metformin, phenformin, hypoxia, microglia, brain ischemia, mitochondrial permeability transition pore, Ca^2+^ signals

## Abstract

Microglial functioning depends on Ca^2+^ signaling. By using Ca^2+^ sensitive fluorescence dye, we studied how inhibition of mitochondrial respiration changed spontaneous Ca^2+^ signals in soma of microglial cells from 5–7-day-old rats grown under normoxic and mild-hypoxic conditions. In microglia under normoxic conditions, metformin or rotenone elevated the rate and the amplitude of Ca^2+^ signals 10–15 min after drug application. Addition of cyclosporin A, a blocker of mitochondrial permeability transition pore (mPTP), antioxidant trolox, or inositol 1,4,5-trisphosphate receptor (IP3R) blocker caffeine in the presence of rotenone reduced the elevated rate and the amplitude of the signals implying sensitivity to reactive oxygen species (ROS), and involvement of mitochondrial mPTP together with IP3R. Microglial cells exposed to mild hypoxic conditions for 24 h showed elevated rate and increased amplitude of Ca^2+^ signals. Application of metformin or rotenone but not phenformin before mild hypoxia reduced this elevated rate. Thus, metformin and rotenone had the opposing fast action in normoxia after 10–15 min and the slow action during 24 h mild-hypoxia implying activation of different signaling pathways. The slow action of metformin through inhibition of complex I could stabilize Ca^2+^ homeostasis after mild hypoxia and could be important for reduction of ischemia-induced microglial activation.

## 1. Introduction

Microglia are the main resident immune cells that are among the first responders to hypoxic/ischemic brain damages [1,2]. Because of their sensitivity to blood flow fluctuations, microglia become activated and undergo morphological changes under hypoxic/ischemic conditions [3]. Microglial activation and thus functions are regulated by a variety of different signals and receptors, such as glutamate [4,5] and adenosine triphosphate (ATP). However, intracellular mechanisms mediating microglial activation under hypoxic conditions are not well understood. When activated, microglia can cause a cascade of inflammatory processes and initiate cytokine release, reactive oxygen species (ROS) production [6]. It is known that intracellular Ca^2+^ signaling is linked with pathophysiological functions of microglia and its signaling changes, arising in response to brain damage, may be activation-associated [7,8,9,10,11]. Previous studies found that calcium signaling is important for microglial immune function—cytokine release [12,13], P2X receptor trafficking and diffusion [14]. Imaging of calcium-dependent fluorescence in vivo has demonstrated that microglial cells have spontaneous Ca^2+^ signaling, but it is quite infrequent in a quiescent state [10,15,16]. However, changes of spontaneous Ca^2+^ signaling are important for microglial function. Signals were changed by Lipopolysaccharide (LPS) [11,16] and in glioma-associated microglia [11]. The rate of signal generation was increased in activated microglia in Alzheimer’s disease mouse models [17], the rate was also elevated by local neuronal tissue injury [18] and aging modified microglial spontaneous Ca^2+^ signaling [19]. Also, changes in neuronal activity triggered increased microglial process Ca^2+^ signaling, which was related to process extension [20]. However, there is still little knowledge on how Ca^2+^ spontaneous signals change in response to hypoxia.

Metformin is widely used as anti-hyperglycemic agent for non-insulin-dependent (type 2) diabetes therapy [21,22,23]. It has been suggested that metformin decreases hepatic glucose production [24] mostly via inhibition of complex I of the mitochondrial respiratory chain [21,25,26] and activation of AMP-kinase (AMPK) signaling pathway [27,28,29]. Recent studies suggest that metformin can also act as a neuroprotective agent during hypoxia/ischemia by suppressing mitochondrial complex I activity in neuronal cultures [30], by reducing ischemic stroke-induced oxidative stress [31,32], inhibiting neuronal apoptosis and suppressing neuroinflammation [33,34]. Phenformin is another biguanide derivative, metformin predecessor with similar chemical structure, which acts as a potential effector of mitochondrial oxidative phosphorylation system inhibiting mitochondrial complex I [35,36]. Both compounds, metformin and phenformin, have also been suggested to inhibit mPTP opening and to exert neuroprotective effects by this mechanism [30]. However, the link between mitochondrial dysfunction and inflammatory responses mediated by the activated microglia are not clear yet, and the role of metformin in these processes remains largely unknown. It has been demonstrated that metformin and phenformin at pharmacologically relevant concentrations differently improve Ca^2+^ homeostasis in hypoxia-affected primary cortical neuronal cell cultures [37], but little is known about their role in microglial cultures.

In this study, we investigated the effect of metformin on spontaneous calcium signals in cultured microglia cells grown under normoxic and mild hypoxic conditions in order to elucidate the mechanism by which ischemic-hypoxic injury induce spontaneous calcium signaling changes in microglia.

## 2. Results

**Figure 1:**
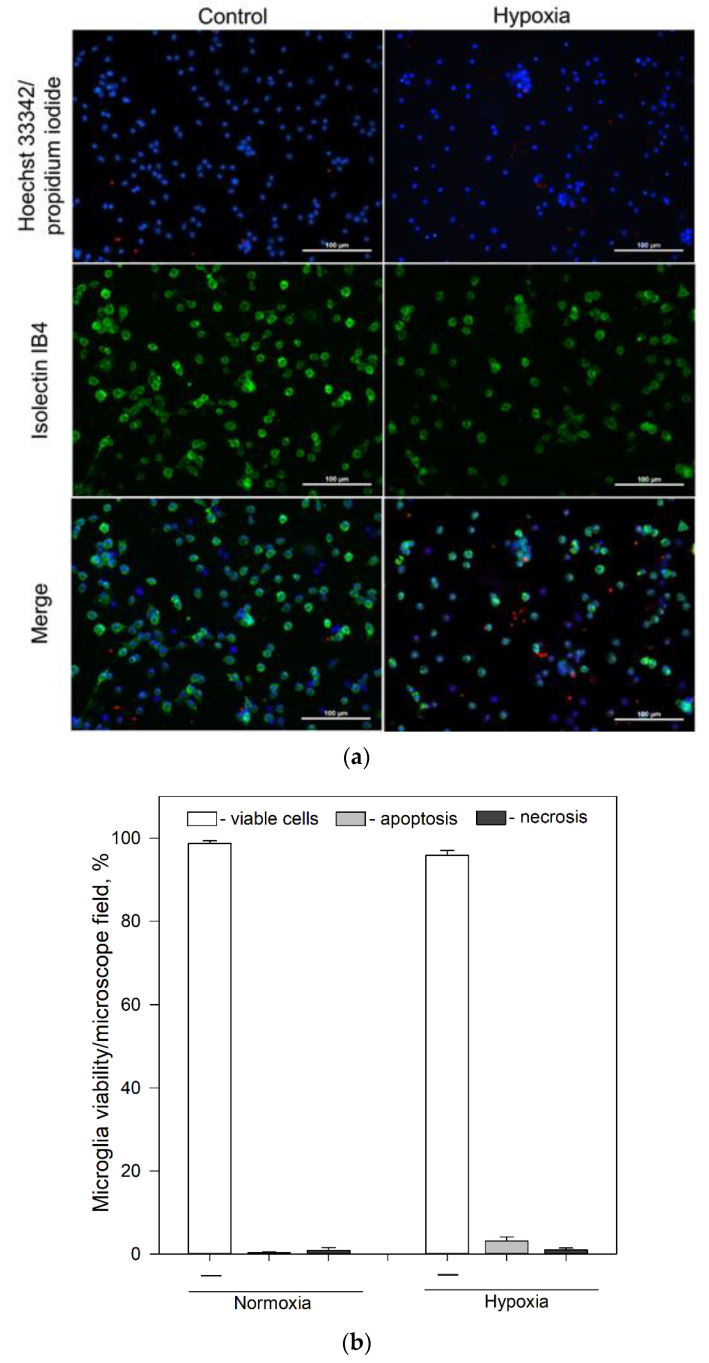
Effect of hypoxia on microglial viability after 24 h incubation. (**a**) Typical fluorescence microscopy images of cultures of microglia under normoxic and hypoxic conditions after 24 h incubation: upper line—control group (normoxia); lower line—microglia after 24 h incubation under hypoxic conditions. First column—cell nuclei labelled with Hoechst33342 (blue) and propidium iodide (red); middle column—microglia labelled with Isolectin IB4 and AlexaFluor488 conjugate (green); third column—merged images. All images were taken at 20× magnification (Olympus IX71, Tokyo, Japan) under identical conditions in each channel and processed identically. The scale bar in the overlay is 100 μm. (**b**) Effects of hypoxia on microglia cell viability after 24 h incubation. Cell viability was assayed as described in Section 4.4. Statistical comparison between the experimental groups were tested for normality using Shapiro–Wilk test and statistically compared between experimental groups by using Student’s *t*-test (Figure 1b). Each bar represents mean ± SE, number of experiments on independent cell culture preparations: N = 16 in normoxia and N = 17 in hypoxia.

Pure microglial cell cultures prepared from 5–7-day-old Wistar rats were used in this study (Figure 1a). Viability of microglia under normoxic (control group) conditions was 98.7% (Figure 1a,b). When cells were exposed to mild hypoxia (2% oxygen) for 24 h a tendency of increased cell death was observed, though viability of microglia still remained above 90% (Figure 1a,b).

**Figure 2:**
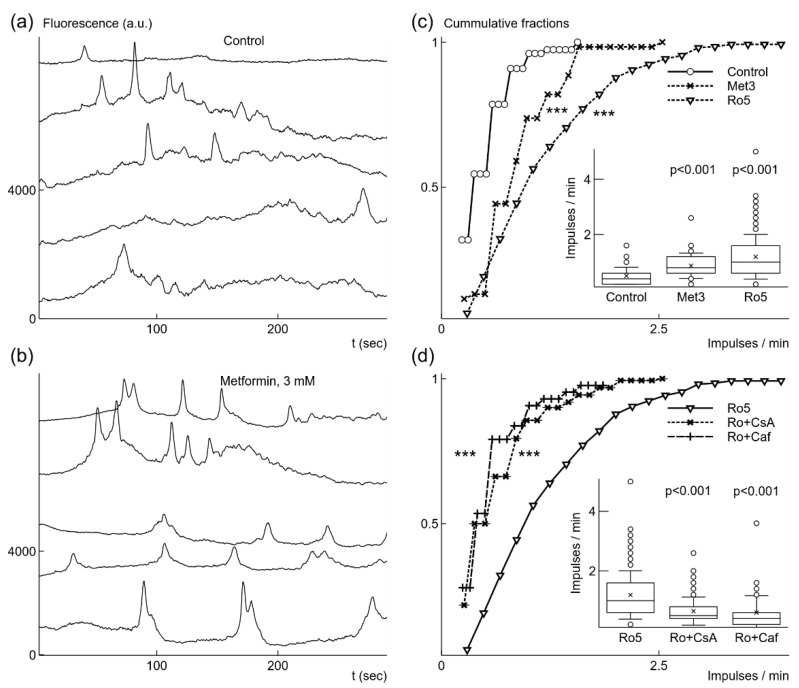
Metformin or rotenone increased the rate of spontaneous generation of fast Ca^2+^ signals under normoxic conditions. In control, Ca^2+^ signals had low frequency, as shown in fluorescence traces from 5 microglia cells recorded simultaneously (**a**). Addition of 3 mM (Met3) metformin caused more frequent signal generation (**b**). The increase in frequency was statistically significant, which is illustrated as both cumulative probability distributions and box-and-whisker plots (label Met3, inset) (**c**). Even stronger increase in the generation rate was evoked by application of 5 nM rotenone (Ro5) (**c**). When 10 μM cyclosporin A, CsA (Ro + CsA) was added in the presence of rotenone the rate was significantly reduced (**d**). Similar reduction of the rate was induced by addition of 5 mM caffeine, Caf (Ro + Caf), (**d**). In box-and-whisker plots error bars indicate 1 standard deviation, shoulders of boxes indicate 25–75% intervals, median of data is highlighted by a horizontal line, mean is indicated by a cross, outliers by circles. For cumulative probability distributions *** corresponds to *p* < 0.001 (Kolmorogov–Smirnov test), *p* from Welch’s *t*-test in box-and-whisker plots. Fluorescence traces were shifted by 2000 a.u. in relation to each other for clarity in (**a**) and (**b**).

Microglia cells generate a variety of different duration Ca^2+^ signals, spontaneous and evoked by stimulation [11,19,38]. We used calcium-dependent fluorescence dye, OGB-AM, to record and analyze generation of spontaneous fast Ca^2+^ signals lasting about 10 or less seconds (Figure 2a) in microglial cultures. In cell cultures grown under normoxic conditions 14.9% of recorded cells (154 out of 1030 in 10 cultures) generated spontaneous Ca^2+^ signals with the relative amplitude ΔF/F = 12.1 ± 5.2%, the half-time T-half = 5.7 ± 1.5 s, and the time of decay Td1/2 = 3.1 ± 1.0 s. The mean generation rate was 0.51 ± 0.31 impulses per minute (imp/min). Similar low spontaneous activity of microglial cells was observed in-vitro and in-vivo previously [11,19].

First, we explored effects of brief exposure to metformin on microglia under normoxic conditions. We used relatively high, 3 mM, concentration of metformin because of its weak and slow inhibition of mitochondrial respiration [25,26]. Application of 3 mM metformin evoked increased spontaneous Ca^2+^ signal generation as observed in fluorescence traces recorded 10–15 min after application (Figure 2b). The generation rate increased by 72% from 0.51 imp/min to 0.87 ± 0.46 imp/min (n = 61, 3 cultures); the increase was statistically significant, as evident from comparison of cummulative fraction distributions (Figure 2c), p < 0.001 (K-S test), and from changes in the means, p < 0.001 (Welch’s t-test) (Figure 2c, inset). Metformin also induced activity in previously silent cells. We recorded spontaneous Ca^2+^ signals in 61 cells out of all recorded 188 microglial cells (3 cultures). The fraction of active cells in the population increased from 14.9% under control, normoxic, conditions to 32.4% after application of 3 mM metformin. In order to check that this effect is due to metformin inhibition of complex I of the respiratory chain in mitochondria [25], we tested canonical inhibitor rotenone [39]. Treatment of microglial cells with 5 nM and concentrations up to 1 µM of rotenone for 24 h had no statistically significant effect on cell viability under normoxic and mild hypoxic conditions (data not shown).

Addition of low concentration, 5 nM rotenone also strongly accelerated generation of Ca^2+^ signals recorded 10–15 min after application. The Ca^2+^ signals were observed in 35.8% of recorded microglial cells (261 out of 729 cells in 20 cultures). Since the part of active cells increased more than 2 times, this means that rotenone also induced spontaneous signal generation. The rate of Ca^2+^ generation increased 2.4 times to 1.2 ± 0.8 imp/min (Figure 2c). The change was statistically significant as it is evident from cumulative distribution, *p* < 0.001 (K-S test), and from changes in the means, *p* < 0.001 (Welch’s *t*-test) (Figure 2c, inset).

Further, we explored what mechanisms could be responsible for spontaneous Ca^2+^ signal generation. Both metformin and rotenone induce generation of ROS [26,40] due to the inhibition of complex I of the mitochondrial electron transfer system. Thus, the observed effects of metformin and rotenone imply a possibility that mitochondrial permeability transition pore (mPTP) could be involved in generation of spontaneous fast Ca^2+^ signals [41,42]. We had chosen to use rotenone as the initial inducer of increased generation of Ca^2+^ signals, and used CsA, trolox, or caffeine as possible modifiers of this induced effect. We did not use metformin for this task because it is a weak inhibitor with slowly evolving effect [25,26]. The application of 10 μM CsA blocking mPTP (Nicolli et al. 1996) reduced 1.8 times the rate of Ca^2+^ signal generation (0.65 ± 0.47 imp/min, *n* = 160, *p* < 0.001) in the presence of 5 nM rotenone (Figure 2d). The number of active microglial cells was also reduced to 19.2% (160 cells out of 865, 10 cultures). Since rotenone induces generation of ROS [40] and mPTP opening is sensitive to increases of ROS [43], we tested Trolox (TRX), a chroman containing antioxidant molecule, which prevents lipid oxidation in mitochondria [44]. The application of 100 μM TRX reduced the rate 2.1 times to 0.57 ± 0.35 imp/min (*n* = 58, *p* < 0.001). The number of active cells was also reduced several times in comparison to activity in the presence of rotenone alone from 35.8% to 12.6% (58 cells out of 461, 6 cultures). Opening of mPTP is sensitive to concentration changes of Ca^2+^ in the cytoplasm mediated by IP3 receptors [41,42]. Thus, we tested sensitivity of spontaneous Ca^2+^ signal generation to caffeine, which blocks IP3R1 [45] known to be expressed in microgial cells [11]. The application of 5 mM caffeine in the presence of 5 nM rotenone decreased the rate 2 times to 0.6 ± 0.57 imp/min (*n* = 43, *p* < 0.001, Figure 2d). The portion of active cells in the population of recorded microglia dropped to 9.3% (43 out of 461, 5 cultures).

**Figure 3:**
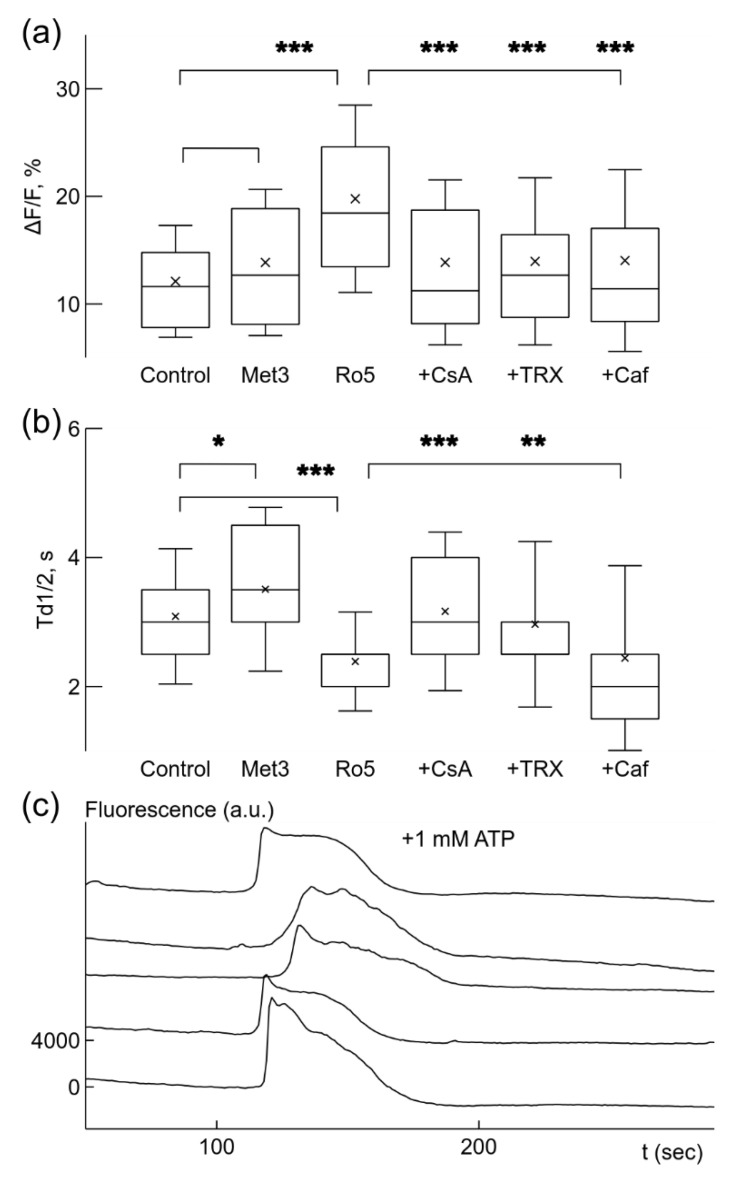
Ca^2+^ signal parameters were changed by drugs under normoxic conditions. Box-and-whisker plots illustrate changes in relative signal amplitude, ΔF/F, (**a**) and the time of decay to half-amplitude, Td1/2, s, (**b**) of spontaneous Ca^2+^ sensitive fluorescence signals in microglial cells. Application of 3 mM metformin (Met3) did not significantly change the relative amplitude (**a**) but increased the time of decay (**b**). Addition of 5 nM rotenone (Ro5) significantly increased the signal strength (**b**) and reduced the time of decay (**b**). Other drugs were added separately when 5 nM rotenone was already present. 10 μM CsA (+CsA) and 100 μM trolox, TRX (+TRX) reduced signal strength (**a**) but increased the time of decay (**b**) comparing to values after rotenone only, while 5 mM caffeine (+Caf) reduced the signal amplitude only (**a**). (**c**) Application of 10 μM CsA together with 5 mM caffeine blocked generation of spontaneous Ca^2+^ signals facilitated by 5 nM rotenone, but addition of 1 mM ATP evoked Ca^2+^-dependent fluorescence changes in simultaneously recorded traces. Note the absence of fast Ca^2+^ signals in the traces. The traces were shifted by 4000 a.u. in relation to each other in (**c**). * *p* < 0.05, ** *p* < 0.01, *** *p* < 0.001 (Welch’s *t*-test).

The observed enhancement of Ca^2+^ signaling due to metformin or rotenone is differently reflected in Ca^2+^ signal parameters, ΔF/F and Td1/2 (Figure 3a,b), calculated for the same set of recorded microglial cells. Application of 3 mM metformin under normoxic conditions did not significantly increase the relative amplitude ΔF/F of the Ca^2+^ signals (Figure 3a). However, this relatively high concentration of metformin, slowed the decay of Ca^2+^ signal, the time of decay to half amplitude, Td1/2, increased 14% from 3.1 ± 1.0 s under control conditions to 3.5 ± 1.3 s (*p* < 0.05, Figure 3b). In contrast, application of 5 nM rotenone strongly increased the relative amplitude, ΔF/F, which was strengthened 1.6 times from 12.1 ± 5.2% in normoxia to 19.8 ± 8.7% (*p* < 0.001, Figure 3a). Instead, the time of decay to half-amplitude, Td1/2, was significantly reduced by 23% from 3.1 ± 1.0 s in normoxia to 2.4 ± 0.77 s (*p* < 0.001, Figure 3b). The effect of rotenone on signal parameters was sensitive to application of CsA, TRX or caffeine. When 10 μM CsA, 100 μM TRX, or 5 mM caffeine were applied in the presence of rotenone, rotenone effect to ΔF/F was significantly reduced by about 30% to values of 13.9 ± 7.7%, 14.0 ± 7.8%, and 14.0 ± 8.4% accordingly (*p* < 0.001 for all changes, Figure 3a). In contrast, the action of these drugs on the time of decay Td1/2 was not uniform. 10 μM CsA and 100 μM TRX significantly increased Td1/2 by 33% to 3.2 ± 1.2 s, and by 24% to 3.0 ± 1.3 s accordingly (*p* < 0.001 and *p* < 0.01, Figure 3b). Application of 5 mM caffeine did not significantly change the time of decay Td1/2 (Figure 3b).

CsA and caffeine act on different mechanisms of Ca^2+^ signal generation—mPTP and IP3R1, accordingly. Since they both reduced the rate of Ca^2+^ signal generation and its amplitude, we checked how these drugs together affected spontaneous rate. Application of 10 μM CsA and 5 mM caffeine in presence of 5 nM rotenone almost completely blocked spontaneous activity in microglial cells, only 2% of all cells in recorded populations remained active (3 out of 148, 5 cultures). In order to be sure that microglial cells remained in a functional state after application of these drugs, we applied 1 mM ATP in order to induce Ca^2+^ concentration changes (Figure 3c). Almost all, 95%, of recorded cells were responsive to 1 mM ATP application (95 out of 100, 4 cultures). These results suggest that spontaneous Ca^2+^ signals are mediated by mPTP and IP3R.

**Figure 4:**
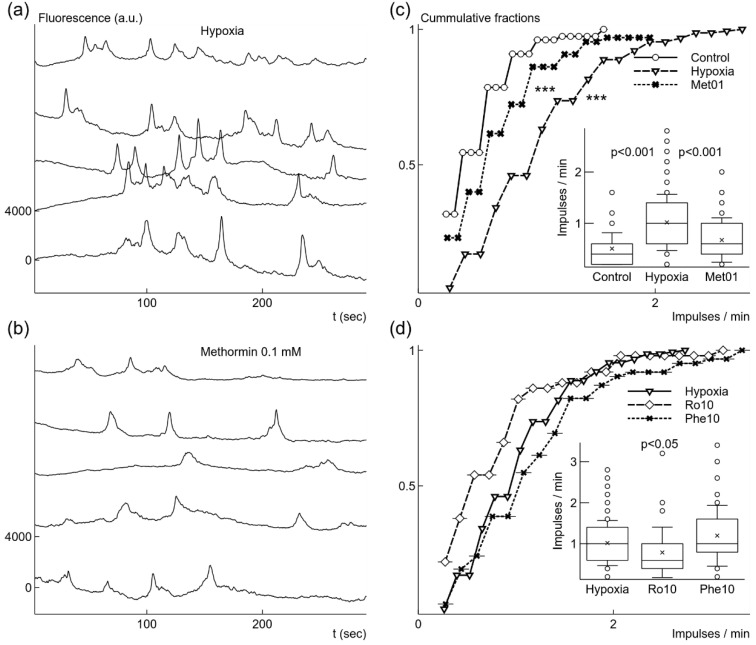
Changes of spontaneous Ca^2+^ signal generation induced by growth under mild hypoxic conditions with or without additional drugs. Microglial cells grown under mild hypoxic conditions showed elevated rate of spontaneous Ca^2+^ signals (**a**). Addition of 0.1 mM metformin before mild hypoxic conditions reduced the rate of Ca^2+^ signal generation (**b**). Elevation of generation rate after hypoxia comparing to cells grown under normoxia was significant as illustrated by cummulative fractions and box-and-whisker plot (**c**). Reduction of generation rate by adding 0.1 mM metformin (Met01) (**c**) was also statistically significant comparing to the rate after mild hypoxia without metformin. 10 nM rotenone (Ro10) added before mild hypoxic conditions weakly reduced the rate of spontaneous signal generation (**d**), although the difference was not statistically significant for cummulative fractions. However, 10 μM phenformin (Phe10) did not change the rate (**d**). Fluorescence traces were shifted by 4000 a.u. in relation to each other in (**a**) and (**b**). *** *p* < 0.001 (Kolmorogov–Smirnov test), *p* from Welch’s *t*-test in box-and-whisker plots.

**Figure 5:**
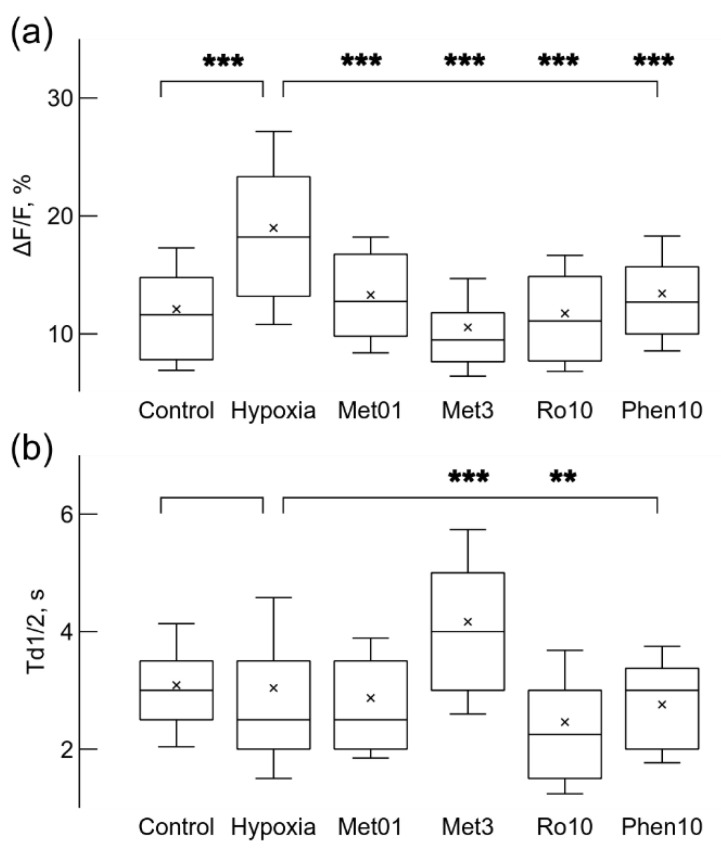
Hypoxia and drugs applied before hypoxic condition changed Ca^2+^ signal relative amplitude, ΔF/F (**a**), and the time of decay to half-amplitude, Td1/2 (**b**). Hypoxia increased the relative strength of Ca^2+^ signals (**a**), while application of 0.1 mM metformin (Met01), 3 mM (Met3) metformin, 10 nM rotenone (Ro10), or 10 μM (Phe10) phenformin reduced the strength when applied separately before growth under mild hypoxic conditions. Only 3 mM metformin significantly increased the time of decay to half-amplitude Td1/2, and 10 nM rotenone reduced it (**b**). ** *p* < 0.01, *** *p* < 0.001 (Welch’s *t*-test).

Next, we explored how growth of microglial cells under mild hypoxic conditions affects spontaneous Ca^2+^ signaling. After hypoxia, microglial cells had higher spontaneous signal generation rate (Figure 4a,c); it increased 2 times from 0.51 imp/min in normoxia to 1.02 ± 0.55 imp/min (*p* < 0.001, *n* = 152 in 4 cultures). Hypoxic conditions induced activity in a large part of cells: the fraction of active cells increased from 14.9% to 40.6% (152 cells out of 374). Ca^2+^ signals also became stronger, the relative amplitude ΔF/F increased by 57% from 12.1 ± 5.2% in normoxia to 18.9 ± 8.2% (*p* < 0.001, Figure 5b); however, the time of decay Td1/2 remained unchanged: 3.0 ± 1.5 s (Figure 5b).

**Figure 6:**
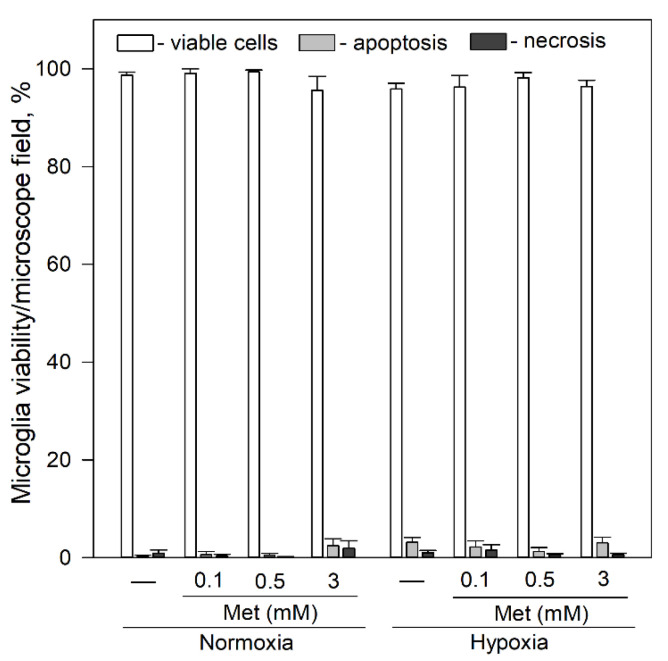
Effects of metformin on microglial cell viability after 24 h incubation under hypoxic and normoxic conditions. Cell viability was assayed as described in Section 4.4. Met—metformin. Statistical comparison between the experimental groups were tested for normality using Shapiro–Wilk test and statistically compared between experimental groups by using One Way ANOVA using Tukey test. Each bar represents mean ± SE (N = 4–17 number of experiments on independent cell culture preparation).

Since metformin is known to affect openings of mPTP [46], we tested its effects on viability and spontaneous Ca^2+^ signal generation in microglia. As shown in Figure 6, exposure of microglia to 0.1–3 mM metformin for 24 h had no significant effect on microglial viability under normoxic and hypoxic conditions.

Then we tested slow action of 30 times reduced, comparing to the experiments in normoxia, 0.1 mM concentration of metformin on spontaneous Ca^2+^ signaling in microglial cells exposed to mild hypoxia. When 0.1 mM metformin was added before putting cells for 24 h under mild hypoxic conditions it had an opposite effect comparing to fast action of 10–15 min application under normoxic conditions. The rate of Ca^2+^ signal generation was substantially reduced by 34% comparing with values obtained after hypoxia alone (Figure 4b,c); the rate was 0.67 ± 0.43 imp/s (*n* = 65, *p* < 0.001, Figure 4d). 0.1 mM metformin also reduced the part of active cells, only 17.2% were active (65 out of 378 cells in 3 cultures). This concentration of metformin also reduced the relative amplitude ΔF/F by 30% from 19% to 13 ± 4.9% (*p* < 0.001, Figure 5b), but it did not change the decay time of the signals (Figure 5b).

Increased concentration, 0.5 or 3 mM, of metformin elicited even stronger effect on Ca^2+^ signaling than 0.1 mM concentration. The rate was reduced by 45% and 63% from 1.02 ± 0.55 imp/min to 0.56 ± 0.42 imp/min (*n* = 61) and to 0.38 ± 0.25 imp/min (*n* = 33) accordingly for 0.5 and 3 mM metformin (*p* < 0.001 for both cases). The fraction of active microglial cells in the population was also reduced to 16.7% (61 out of 364, 3 cultures), and to 7.8% (33 out of 421 cells in 4 cultures) accordingly for 0.5 and 3 mM concentrations. These concentrations of metformin also had stronger effect than 0.1 mM metformin on the relative amplitude ΔF/F, which was reduced by 20% and 44%, from 19.0 ± 8.2% to 15.2 ± 6.7 % and to 10.6 ± 4.1% (*p* < 0.001 in both cases) accordingly for 0.5 and 3 mM (Figure 5b) concentrations. Although 3 mM metformin showed a more desirable, stronger, reduction on the rate and the strength of signals, it increased the time of decay Td1/2, i.e., broadened the Ca^2+^ signal, by 37% from 3.0 ± 1.5 s to 4.1 ± 1.7 s (*p* < 0.001, Figure 5b).

In order to check whether the observed effect of metformin is due to inhibition of complex I, we tested a canonical inhibitor rotenone by applying 2 times stronger, comparing to experiments under normoxia, 10 nM concentration before mildly hypoxic conditions lasting 24 h. The application reduced the rate of spontaneous signal generation by 22% from 1.02 ± 0.55 imp/min to 0.79 ± 0.61 imp/min (*n* = 50, Figure 4b). However, the difference was significant only in Welch’s *t*-test (*p* < 0.05), but K-S test did not show significance for differences between probability distributions (Figure 4b). The fraction of active cells was reduced from to 40.6% to 20.1% (50 cells out of 249 in 4 cultures). The signal parameters were also affected, the relative amplitude reduced by 38% from 19% to 11.7 ± 4.9% (*p* < 0.001), while the time of decay was reduced by 19% from 3.0 ± 1.5 s to 2.4 ± 1.2 s (*p* < 0.01). The increased 50 nM concentration of rotenone had stronger effects. The generation rate was reduced by 38% to 0.62 ± 0.45 imp/min (*n* = 52, *p* < 0.001), the relative amplitude ΔF/F was reduced by 33% to 12.5 ± 4.5% (*p* < 0.001), the time of decay Td1/2 was reduced by 24% to 2.3 ± 1.0 s (*p* < 0.001), and the fraction of active cells was only 16.5% (52 cells out of 315 in 4 cultures). These results show that rotenone after 24 h induced similar effects to metformin, except for the time of decay, for which the reduction was also observed when rotenone was applied under normoxic conditions. Since both metformin and rotenone reduced Ca^2+^ generation rate and signal strength, this means that the prolonged blockade of complex I lasting 24 h under mild hypoxic conditions induced opposite effects compared to the fast action of these drugs after short 10–15 min application under normoxic conditions.

To compare slow metformin effects with action of similar biguanides, we used phenformin, which like metformin blocks mitochondrial respiration by acting on complex I [36]. Incubation of microglia with 10–25 μM phenformin for 24 h under normoxic or mild hypoxic conditions had no effect on cell viability (data not shown).

Then we looked into whether phenformin affects Ca^2+^ signaling when added before hypoxia. Surprisingly, 10 μM concentration phenformin did not change the rate of spontaneous Ca^2+^ signals (Figure 4d), but it reduced by 29.2% the relative amplitude ΔF/F to 13.4 ± 4.9% (*n* = 62, *p* < 0.001, Figure 5a). The fraction of active microglial cells in the population was reduced from 40.6% to 30.5% (62 out of 203 cells in 3 cultures). Phenformin was unable to reduce the rate of spontaneous Ca^2+^ signals even at higher concentration of 25 μM, the rate was 1.05 ± 0.74 imp/s (*n* = 103). The relative amplitude ΔF/F was reduced by 24.2% to 14.3 ± 6.8% (*p* < 0.001), i.e., the reduction did not become stronger with elevation of concentration. Even worse, this concentration of phenformin broadened the signal, Td1/2 was increased by 19.0% to 3.62 ± 2.04 s (*p* < 0.05). The portion of active cells was 41.4% (103 out of 249 cells in 4 cultures), i.e., it stayed almost the same as after mild hypoxia without drugs.

## 3. Discussion

We studied spontaneous Ca^2+^ signals in microglial cells. The first novel finding of this study was that metformin and low, 5 nM, concentration of rotenone 10–15 min after application induced elevated generation rate of Ca^2+^ signals in cells grown under normoxic conditions, while addition of CsA, or TRX, or caffeine reduced the elevated rate, suggesting that mPTP together with IP3R generate and mediate spontaneous Ca^2+^ signals. The second finding was that microglial cells grown under mild hypoxic conditions for 24 h showed elevated rate of spontaneous Ca^2+^ signals. The treatment before mild hypoxia with low concentration of metformin (0.1 or 0.5 mM), or rotenone (10 or 50 nM), but not phenformin (10 or 25 μM), reduced this elevated rate.

In experiments under normoxic conditions, we registered an increased spontaneous Ca^2+^ signal generation after 10–15 min application of 3 mM metformin. We used a relatively high concentration of metformin, since this drug is known to be weak inhibitor of complex I in mitochondrial respiratory chain with IC50 value from 20 mM to 79 mM [25,26]. We recorded Ca^2+^-dependent fluorescence traces 10–15 min after application because metformin action was shown to be very slow in previous experiments [25,26]; it took almost 200 min to block 50% of respiration rate by 10 mM metformin in isolated rat liver mitochondria [25]. Although the effect of metformin on mitochondrial respiration is weak and slow, it still can produce deleterious action on the cell, since metformin induces ROS production by the complex I flavin [26]. Thus, if ROS production is regulating spontaneous Ca^2+^ signaling, the observed increased spontaneous Ca^2+^ activity in our experiments could be quite expected. In order to check that this effect is brought about by action of metformin only on complex I, we tested a canonical complex I inhibitor rotenone, which blocks NADH oxidation by mitochondria [39], and its evoked neuronal degeneration is thought to be mediated by microglial NADPH oxidase [47]. Rotenone also has much faster inhibitory effect on mitochondrial respiration comparing to metformin or phenformin [26].

We observed increased rate of spontaneous Ca^2+^ signals after 10–15 min application of 5 nM rotenone under normoxic conditions. The concentration used was chosen in order to avoid substantial disruption of cell respiration but was still to be able to induce elevated ROS production. It was shown in previous studies that 10 nM rotenone reduced respiration by 30% and induced noticeable generation of H_2_O_2_ [40]. Assuming that spontaneous Ca^2+^ signals were mediated by mPTP, it would be expected that increased ROS production elevated the rate of mPTP opening [43] and hence the Ca^2+^ signal generation. In support of this assumption, ROS elevation in astrocytes enhanced mPTP-dependent Ca^2+^ release from mitochondria in spontaneous Ca^2+^ signals [42]. Increased probability of mPTP opening could also explain enhanced amplitude of Ca^2+^ signals by rotenone application. Although we used only 5 nM concentration or rotenone, its effect on spontaneous Ca^2+^ signaling was much stronger comparing to 3 mM metformin: the generation rate was higher, and the strength of the signals increased. Thus, weak and slow action of metformin is much more sparing for the microglial cells under normoxic conditions. It should be noted that the observed effect of enhanced signaling due to 5 nM rotenone stands in contrast to its blocking effect induced by higher micromolar concentrations as it was shown on mPTP mediated Ca^2+^ signals in astrocytes [42] and mPTP mediated ROS flashes in HeLa cells [48]. However, such micromolar concentrations should had completely blocked mitochondrial respiration [40].

In order to elucidate the mechanisms of regulation of spontaneous Ca^2+^ signaling we used different drugs in the presence of 5 nM rotenone which elevated the signal generation rate. This higher spontaneous signal rate allowed us to detect reduction of spontaneous activity more easily than control, normoxic, conditions with low activity. Since both metformin and rotenone, induce production of ROS [26,40,49], we tested hypothesis that spontaneous Ca^2+^ signals were mediated by mPTP. We observed that 10 μM CsA, which blocks mPTP [50], strongly reduced the rate of Ca^2+^ signals and their amplitude. Also, CsA prolonged the time of decay to half-amplitude Td1/2 and reduced the number of active microglial cells almost two times. Such effects show that CsA reduced the probability of opening of channels mediating Ca^2+^ flux to the cytoplasm, corroborating the hypothesis that observed Ca^2+^ signals were mediated by mPTP. The concentration of CsA used in our experiment should had been enough to strongly or completely block openings of mPTP, since 2 μM CsA reduced the rate of mPTP mediated ROS production flashes several times in HeLa cells [48], and in isolated mitochondria IC50 for mPTP blockage was only 40 nM [50]. However, we observed only reduction of activity but not complete elimination of spontaneous signaling. The only partial blocking effect of CsA could be explained either by the lack of cyclophilin D, as for example in astrocytes [42], or by influence of other proteins influencing the opening of mPTP.

Since 5 nM rotenone increased the rate of Ca^2+^ signals presumably due to ROS generation, it was unsurprising that 0.1 mM Trolox (TRX), a chroman containing antioxidant molecule, reduced the rate by several times. The concentration of 0.1 mM used in our experiments is sufficient to reduce lipid oxidation in mitochondria by 60% [44]. Since the relative amplitude ΔF/F was also reduced by 30% and the number of microglial cells in the population was cut several times, this effect corroborates ROS influence on Ca^2+^ signaling probably by affecting mPTP openings.

In previous studies, it was shown that spontaneous Ca^2+^ signals in microglial cells depend on IP3 receptors [11]. We checked whether this effect is present in our case by applying 5 mM caffeine in presence of 5 nM rotenone. Such concentration should completely block openings of IP3R1 channels [45], since concentration for half-inhibition was found to be 1.6 mM [51], and 5 mM completely blocked responses to IP3 in *Xenopus* oocytes [52]. However, in our recordings, such application of caffeine in the presence of rotenone caused only reduction, not complete blockade, of the rate, relative amplitude, and the number of active cells in the population. Although, the time of decay to half-amplitude of Ca^2+^ signals was not affected. This result suggests that IP3R1 is involved in generation of spontaneous Ca^2+^ signals in microglial cells. The partial blocking effect on spontaneous Ca^2+^ signals of the application of caffeine could suggest that IP3R2 or IP3R3 [11], which are not affected by caffeine [45], influence spontaneous Ca^2+^ signaling. However, application of CsA together with caffeine in the presence of rotenone completely blocked spontaneous signals. Such blocking was not related to deterioration of the cells since application of 1 mM ATP under such conditions evoked Ca^2+^ response (Figure 3c) in almost all recorded microglia cells.

Apparently, spontaneous Ca^2+^ signals are mediated by mPTP interacting with IP3 receptors. It is well known that IP3 receptors are involved in endoplasmic reticulum-mitochondrial contacts [53] and mediate Ca^2+^ transfer to mitochondria [54]. Interaction between mPTP and IP3R was observed in evoked, so-called Ca^2+^-induced release of Ca^2+^ from mitochondria (mCICR), responses [41]. Openings of mPTP, sensitive to CsA, amplified evoked Ca^2+^ responses in mCICR [41]. In agreement, in our study we saw that CsA and caffeine affect the rate and relative amplitude of the spontaneous Ca^2+^ signals. A similar interaction between mPTP and IP3R in generation of spontaneous Ca^2+^ signals was observed in astrocytes: CsA with micromolar concentration of rotanone reduced the frequency and the amplitude of the signals, while the generation was weakened in IP3R2–/– mice [42]. In our experiments were measured influence of drugs on the time of decay Td1/2, which was influenced by agents acting on mPTP openings—rotenone, CsA and Trolox, while caffeine did not influence this signal parameter. This result suggests that opening of mPTP, but not IP3R, defines the duration of evoked Ca^2+^ fluxes.

What are the possible mechanisms of spontaneous Ca^2+^ signal generation in microglial cells? mPTP openings during induced mCICR were triggered by elevation of pHi inside mitochondria [41]. Spontaneous openings of mPTP also induced flashes of ROS generation in mitochondria, so-called mitoflashes [55,56], which were triggered by changes in mitochondrial pHi and occurred completely randomly [57], which explains their spontaneous nature. It was shown that such mitoflashes are universal feature of mitochondria and were observed in different types of cells [55,56]. Such mechanisms could be employed in microglial cells, since spontaneous Ca^2+^ signals were completely blocked by FCCP [11], which eliminates proton gradient by transporting protons through the membrane [58]. Thus, it is possible that the same mechanisms of pHi-dependent triggering could explain spontaneous nature of observed Ca^2+^ signals in our recordings, since the observed rate of 0.5 imp/min under normoxic conditions is similar to that reported in other studies. The rate of spontaneous flashes of ROS generation governed by openings of mPTP was 0.3 imp/min [48] in mouse embryonic fibroblasts. In astrocytes, the rate of spontaneous Ca^2+^ signals in separate domains co-localized with mitochondria and mediated by mPTP was 0.4 imp/min [42].

In microglial cells grown under 24 h mild hypoxic conditions we observed elevated rate of spontaneous Ca^2+^ signal generation, stronger amplitude and larger number of active microglia in the population of recorded cells comparing to the ones grown under normoxic conditions. Such effects were similar to the changes in spontaneous Ca^2+^ signaling evoked by 10–15 min application of metformin or low rotenone concentration under normoxic conditions. It is possible that they are due to the same mechanism of elevated ROS acting on mPTP openings, since after growth of microglial cells under mild-hypoxic conditions for 24 h, we made recordings under normoxic conditions, which induced mild reoxygenation effect known to cause ROS production [59]. In support of this possibility, spontaneous flashes of ROS generation dependent on mPTP openings had increased rate during early phases of reoxygenation [55]. According to [60] elevated mitoflash activity reflects increased ATP synthesis and serves as negative feedback in order to restore it to the functionally required state. This suggests that under our experimental conditions mild reoxygenation experienced by microglial cells after hypoxic conditions upregulates ATP production and probably, as a result, increases microglial activity.

We tested metformin action on spontaneous Ca^2+^ signals in microglial cells grown under mild hypoxic conditions for 24 h, where metformin was added before application of hypoxia. Differently from metformin action after 10–15 min in normoxia, all concentrations tested (0.1, 0.5 and 3 mM) after 24 h in mild-hypoxia-induced reduction of the spontaneous Ca^2+^ signal generation rate. This reduction increased with metformin’s concentration. This result could be expected since metformin ability to inhibit openings of mPTP were shown previously in ischemic heart and isolated heart mitochondria [46,61]. The relative amplitude of the signals was also reduced reflecting lower opening probability of mPTP. The largest reducing effect was for 3 mM metformin; however, this concentration also increased by 37% the time of decay Td1/2, although neither hypoxia alone, nor 0.1 or 0.5 mM metformin changed this parameter. This broadening of the spontaneous Ca^2+^ signals possibly reflects deterioration of Ca^2+^ homeostasis of the cells due to the stronger effect of 3 mM metformin on mitochondrial respiration [62]. The counteracting action of metformin acting on the signal generation rate increased by 24 h hypoxia could be mediated by activation of PI3K [46], or AMPK [60,63], or HIFα [49]. It should be noted that these signaling pathways require at least several hours to be activated and to bring about their effects [46,49,60]. These longer effects of metformin application due to induced ROS generation [49] could explain the difference between the short effect of strengthening of spontaneous Ca^2+^ signaling when metformin was applied 10–15 min in normoxia, and the slow inhibitory effect when metformin was applied before mild hypoxia for 24 h.

Metformin evoked inhibition of spontaneous Ca^2+^ signal generation after 24 h mild hypoxia was due to inhibition of complex I because application of rotenone 10 or 50 nM instead of metformin also caused inhibition of spontaneous Ca^2+^ signaling in our experiments. Like metformin applied for 24 h, rotenone reduced the generation rate, the relative amplitude of Ca^2+^ signals, and the fraction of active cells in the recorded population of microglial cells. Such effects of long duration rotenone application could be explained by reduction of ROS generation and suppressed openings of mPTP, as it was observed in cortical and cerebellar mitochondria [64]. This inhibitory effect was also slow, like in our experiments, it was observed 2 h after application and ischemia [64]. The similarity of slow inhibitory effect evoked by metformin and rotenone applied before 24 h mild hypoxia on Ca^2+^ signaling implies that metformin action is due to complex I blockade and its initial induction of ROS generation.

We also tested effect of long-lasting application of another biguanide, phenformin, which is more potent than metformin in inhibiting cellular oxygen consumption [62], on the spontaneous Ca^2+^ signaling when added before 24 h mild-hypoxia. Surprisingly, neither 10 nor 25 μM phenformin reduced the rate of signal generation, although the fraction of active cells was reduced at lower concentration of phenformin. The higher concentration of phenformin increased the time of decay Td1/2 suggesting deterioration of Ca^2+^ homeostasis brought about by inhibition of respiration. Such effects on the Ca^2+^ signaling are in stark contrast to metformin action. There could be several explanations for this difference. Metformin, but not phenformin, suppresses energy transduction by selectively inducing a state in complex I where redox and proton transfer domains are no longer efficiently coupled [62]. Also, metformin action could be self-limiting and transient [65], because metformin, but not phenformin, self-limits its entry into mitochondria due to its mitochondrial accumulation dependency on membrane potential [25]. This also implies much slower action on respiratory chain of metformin comparing to phenformin and rotenone [25,26].

Inhibition of complex I in mitochondrial respiration chain leads to ROS generation and lack of ATP which could damage the cell. Slower initial induction of metformin effects could protect the cells from such fast deleterious effects by enabling adaptation through a number of slow signaling pathways, AMPK, PI3K, or HIF1α. In our experiments, we saw such initial and possibly damaging action of blocking complex I, which was different for metformin and rotenone. Application of 5 nM rotenone under normoxic conditions after 10–15 min caused relatively much stronger increase in spontaneous Ca^2+^ signal generation comparing to 3 mM metformin. In contrast, ‘slow’ and suppressing effect of 3 mM metformin applied before 24 h mild hypoxia was relatively much stronger comparing to ‘slow’ suppressing rotenone effect. Although rotenone had 2 times larger concentration than that applied in normoxia. Since biguanide uptake into mitochondria is slow and protein-mediated [36], the fast-damaging effect of metformin application could be reduced and slow suppressing effect on spontaneous Ca^2+^ signaling could become stronger comparing to passively diffusing and fast-acting rotenone. Thus, slow and self-limited action of metformin [65] is a beneficial property allowing preservation of Ca^2+^ homeostasis. Since Ca^2+^ signaling is involved in microglial immune functions [12] and in reaction to injury [16], metformin could preserve neuronal tissue after ischemia much better than rotenone or phenformin.

In this study, we explored only spontaneous Ca^2+^ signaling in cultured microglia cells isolated from young animals. Future research could aim at elucidating metformin-induced changes in function of microglia in the brain of adult animals affected by ischemia or injury. Metformin different short- and long-term action on microglia activation could be important for its clinical application. Since metformin potentially could activate microglia during the first hour of application, it could be damaging to nervous tissue if applied directly after injury or ischemia. Alternatively, metformin could have preserving action if taken in advance in case of risk of blood supply during planned brain surgery or increased risk of ischemic stroke.

## 4. Materials and Methods

### 4.1. Reagents and Materials

Reagents for pure microglial cultures preparation and maintaining were obtained from Gibco-Invitrogen (Paisley, UK). Poli-(L)-lysine (Cat# 3438, Minneapolis, MN, USA) was from R&D Systems, isolectin GS-IB4 from Griffonia simplicifolia Alexa Fluor 488 Conjugate (Cat# I21411, South San Francisco, CA, USA) and Oregon Green 488 BAPTA-1,AM (OGB-AM) (Cat# 06807, Bleiswijk, The Netherlands) from Molecular Probes, Thermo Fisher Scientific. Phenformin (Cat# P7045, Sigma–Aldrich, St Louis, MO, USA) and Metformin (Cat# PHR1084, Sigma–Aldrich, St Louis, MO, USA) hydrochlorides, Cyclosporin A (Cat# 30024, St Louis, MO, USA), Rotenone (Cat# R8875, Sigma–Aldrich, St Louis, MO, USA), Caffeine (Cat# C0750, Sigma–Aldrich, St Louis, MO, USA), Trolox (Cat# 238813, Sigma–Aldrich, St Louis, MO, USA) and all others chemical for solutions were purchased from Sigma–Aldrich (St Louis, MO, USA), unless stated otherwise.

Metformin and phenformin was dissolved in double-distilled water (ddH_2_O). CsA, Ro and Trolox was dissolved in ethanol, final concentration of ethanol in cell culture medium do not exceed 0.2% and 0.5% (CsA and Ro, respectively) or 0.67% (Trolox). Caffeine was dissolved in buffer containing NaCl (140 mM), KCl (5.4 mM), (0.98 mM) MgCl_2_ * 6 H_2_O, (1.97 mM) CaCl_2_ * 2 H_2_O, (6.08 mM) HEPES, (9.9 mM) glucose with slight heating (37 °C). Oregon Green 488 BAPTA-1,AM (OGB-AM) was prepared in DMSO (100 µM).

### 4.2. Pure Microglial Culture Preparation

Primary pure microglial cultures from rat cerebral cortices were prepared from 5–7-day-old Wistar rats pups (both genders). Experimental procedures involving animals were undertaken in accordance with the EU Directive 2010/63/EU for animal experiments and the Republic of Lithuania law on the care, keeping, and use of experimental animals (Approved by Lithuanian State Food and Veterinary Service, ethical approval No. B6 (1.9)-855). The rat pups were bred and maintained at the Lithuanian University of Health Sciences animal house under controlled conditions. Pups were euthanized by CO_2_ and decapitated. Then the brain was removed and placed in a Petri dish containing 4 °C phosphate-buffered saline (PBS) supplemented with 1% penicillin-streptomycin. After removal of meninges and blood vessels, cortices was dissociated in Versene (1:5000) for 5 min and centrifuged at 290× *g* for 5 min. Then cells were resuspended in the fresh DMEM-GlutaMax culture medium supplemented with 10% fetal bovine serum and 0.1% penicillin-streptomycin and filtered twice through 100-μm and 40-μm pore-size cell strainers (Corning, Merck). Cells were maintained in the fresh culture medium and grown in a humidified 37 °C incubator with 5% CO_2_. Microglial cells were plated in T75 flasks coated with 0.0005% poly-L-lysine. After 24 h after initial seeding, medium was changed with fresh media and thereafter every two days one-half of the medium was replaced with fresh, pre-warmed culture media. Cell were cultured for 7–11 days in vitro (DIV) after initial seeding. Pure microglial cell cultures were obtained by mechanically shaking flasks. Then medium with detached cells was centrifuged, cells suspended in fresh growth medium and plated at 100,000 cells/mL density into 96-well plates (for viability assay) and into 6-well plates (for Ca^2+^ Assessment) pre-coated with 0.0005% poly-L-lysine solution. The trypan blue dye exclusion test was used to determine the number of viable cells present in a cell suspension after cell detachment. The cultures were maintained at 37 °C with 5% carbon dioxide (CO_2_) for 24 h to allow the cells to attach. These cultures were used for experiments. The purity of the cultures was ≥85%, as assessed by cellular morphology.

### 4.3. Experimental Model of Hypoxia and Cell Culture Treatments

For cell viability assays the cultures were treated for 24 h with metformin (Met 0.1 mM, 0.5 mM and 3 mM) and phenformin (Phen 10 µM, 25 µM) under normoxic or hypoxic (2% O_2_) conditions. To induce hypoxia microglial cell cultures were exposed to hypoxic conditions by keeping microglia cells in humidified chamber (93% N_2_, 5% CO_2_, 2% O_2_) at 37 °C. In normoxic conditions, cell cultures were incubated with or without pharmacological agents in a humidified incubator (5% CO_2_, 37 °C) for 24 h. Cells incubated under normoxic conditions were evaluated as a control group.

To investigate the effect of Met and Phen during calcium-dependent fluorescence measurements in live cells, microglia were incubated under hypoxic and normoxic conditions for 24 h.

To analyze calcium-dependent signals in real-time, cell culture media was supplemented with cyclosporin A (CsA 10 µM), rotenone (Ro 5 nM), trolox (0.1 mM), and caffeine (Caf 5 mM). During calcium-depended fluorescence measurements of Caf, cells were kept in standard buffer as described by [11].

### 4.4. Cell Viability Assay

The viability of microglial cells in cultures was assessed by propidium iodide (PI, 7 μM) and Hoechst 33342 (4 μg/mL) staining using a fluorescence microscope (Olympus IX71, Tokyo, Japan). PI-positive cells were considered to be necrotic and cells stained with cell-permeable DNA dye Hoechst 33342 and showing chromatin condensation/fragmentation but lacking PI staining were classified as apoptotic cells. PI-negative cells with weak Hoechst staining were considered to be viable [66,67]. Microglial cells were identified, and their number were monitored using fluorescent dye Isolectin GS-IB4 conjugated with Alexa Fluor 488 (7 ng/mL, Invitrogen). Number of microglia in cultures were assessed by counting microglial cells in at least 5 random microscopic fields/well and expressed as percentage of specific microglial cells of total number of microglia cells per field. Data were analyzed with ImageJ program.

### 4.5. Ca^2+^ Assessment

Half of conditioned growth medium was removed and 2 µM Green 488 BAPTA-1 was added to cell cultures. After incubation for 10 min (37 °C), cell medium was changed to conditioned growth medium and additionally incubated 10 min (37 °C) before measurements. Ca^2+^ assessment was carried out using 6-well plates.

Imaging was performed with an Andor Neo high-resolution (5.5 Mpixel) sCMOS video camera (Andor Technology Ltd., Belfast, UK). Fluorescence images were obtained by illuminating with a high power 480 nm LED light source (Prizmatix Ltd., Givat-Schmuel, Israel) via U-MWIBA3 band pass 510–550 nm filter (Olympus Corporation, Tokyo, Japan). The LED light was limited to the recording site by a diaphragm. To limit photo-damage of the cells the recording site was used to acquire fluorescence traces only once. Images were acquired with Solis software (Andor Technology Ltd., Belfast, UK) and stored on the disk for further analysis. Image analysis was performed with Solis analysis software, and by employing image analysis package ImageJ, and custom written subroutines.

### 4.6. Fluorescence Analysis

Cultured cells stained with calcium sensitive dye OGB-AM were registered in an area of 1280 × 500 pixels. For analysis 2 × 2 camera pixels were binned for each image pixel in order to reduce noise. In every culture, 3 to 5 different regions were registered. Fluorescence images were acquired during 300 s with the rate of 2 Hz. In order to identify the cells with significantly large changes in calcium concentration, image stacks were analyzed by custom written Python subroutines by using SciPy open source software package. The fluorescence traces from single pixels were selected if the peaks were larger than 5% of the time-averaged fluorescence and the amplitude of the peak was 5 times larger than the smallest standard deviation in pixels of the image stack. Since each separate cell was imaged by a number of pixels, the detected signals were summed and averaged if they were all present in neighboring pixels in the area smaller than 20 × 20 pixels. The areas with the detected signals in the image stacks were also inspected by using ImageJ2 software package. The time course of the detected fluorescence signals was corrected for bleaching by Simple Ratio Method [68]. In this procedure, the decaying mean intensity of fluorescence in the region outside the detected signals was used to calculate the decay ratio for each image and to compensate for the bleaching in the time course of the signals. The traces of signals were used to measure the resting fluorescence level, R, estimated just before the spontaneous change of calcium concentration, and the peak fluorescence value, A. For analysis of signal strength and frequency of its generation, we used the relative peak fluorescence values ΔF/F, A divided by R, only if they exceeded 5% threshold. For analysis of signal duration and its decay in a cell, the half-time, T-half, was measured as duration at the level of half-amplitude, and the time of decay, Td1/2, from the peak to the level of half-amplitude. In the fluorescence trace, the parameters ΔF/F, T-half, and Td1/2 were evaluated for the signal with the highest amplitude.

### 4.7. Statistical Analysis

Data on cell viability expressed as mean ± SE of at least 3 experiments on separate cell culture preparations. Statistical comparison between the experimental groups was performed by Student’s *t*-test and by multiple comparisons using one-way ANOVA, Tukey test using SigmaPlot 12.0 software. *p* < 0.05 was considered significant. Normality of fluorescence data distribution was assessed by using the Shapiro-Wilk test. The statistical significance of the difference between averages was assessed using independent two-sample Welch’s *t*-test for normally distributed data. Statistical significance between cumulative probability distributions was also assessed using the Kolmogorov–Smirnov (K-S) test. All statistical analyses were conducted using procedures from SciPy package.

## Data Availability

The data that support the findings of this study are available from the corresponding author upon request.
